# Cellular and Genetic Analysis of Wound Healing in *Drosophila* Larvae

**DOI:** 10.1371/journal.pbio.0020239

**Published:** 2004-07-20

**Authors:** Michael J Galko, Mark A Krasnow

**Affiliations:** **1**Howard Hughes Medical Institute and Department of BiochemistryStanford University School of MedicineStanford, CaliforniaUnited States of America

## Abstract

To establish a genetic system to study postembryonic wound healing, we characterized epidermal wound healing in *Drosophila* larvae. Following puncture wounding, larvae begin to bleed but within an hour a plug forms in the wound gap. Over the next couple of hours the outer part of the plug melanizes to form a scab, and epidermal cells surrounding the plug orient toward it and then fuse to form a syncytium. Subsequently, more-peripheral cells orient toward and fuse with the central syncytium. During this time, the Jun N-terminal kinase (JNK) pathway is activated in a gradient emanating out from the wound, and the epidermal cells spread along or through the wound plug to reestablish a continuous epithelium and its basal lamina and apical cuticle lining. Inactivation of the JNK pathway inhibits epidermal spreading and reepithelialization but does not affect scab formation or other wound healing responses. Conversely, mutations that block scab formation, and a scabless wounding procedure, provide evidence that the scab stabilizes the wound site but is not required to initiate other wound responses. However, in the absence of a scab, the JNK pathway is hyperinduced, reepithelialization initiates but is not always completed, and a chronic wound ensues. The results demonstrate that the cellular responses of wound healing are under separate genetic control, and that the responses are coordinated by multiple signals emanating from the wound site, including a negative feedback signal between scab formation and the JNK pathway. Cell biological and molecular parallels to vertebrate wound healing lead us to speculate that wound healing is an ancient response that has diversified during evolution.

## Abstract 2

A powerful new system for studying wound healing in the fruitfly is helping to unearth the genetic and cellular requirements of the healing process

## **Introduction**

The capacity to heal wounds is essential for organisms to endure and thrive despite an occasionally hostile environment. Organisms throughout the animal kingdom can heal wounds, but mammalian wound healing has been studied most intensively because of its medical relevance. Wound healing must occur to restore health after trauma or surgery, or in conditions such as cancer or peptic ulcers in which internal processes cause tissue damage. Mammalian epithelial tissues display a characteristic set of responses to tissue damage, including the rapid formation of a blood clot at the site of injury, followed by spreading of the damaged epithelium across the wound gap to restore tissue integrity (Martin 1997; Singer and Clark 1999). However, there are significant differences in the wound healing response depending on the specific tissue affected, its developmental stage, and the nature of the damage. For example, damaged fetal epidermis heals without leaving a scar (Colwell et al. 2003), and a few adult tissues, including human liver, can regenerate large portions of the damaged tissue (Diehl 2002). Some wounds, such as the common foot ulcers of diabetics, heal slowly or not at all (Greenhalgh 2003), whereas others display an exaggerated response that results in disfiguring keloid scars (Alster and Tanzi 2003). One important goal of wound healing research is to find ways to speed or alter the healing process. Another is to understand the fundamental cellular and molecular mechanisms by which cells sense tissue damage and signal to neighboring healthy cells to contain and repair it.

Cellular studies of mammalian wound healing have shown that it is a complex process that takes weeks to complete and involves not just the damaged epithelial cells and their neighbors, but also fibroblasts and blood vessels in the underlying stroma, and inflammatory cells that are recruited to the wound site (Martin 1997; Singer and Clark 1999). Only the first step in mammalian wound healing, the proteolytic cascade that culminates in fibrin deposition and clot formation, is well understood at the molecular level (Furie and Furie 1992). As the clot forms, platelets bound to it and to the damaged tissue release additional procoagulant proteins as well as growth factors and chemokines that can attract neutrophils and monocytes that mediate an early inflammatory response. Keratinocytes at the wound margin become activated, break down their cell junctions, and assume a lamellipodial crawling morphology as they spread across the wound site to restore epithelial integrity (Odland and Ross 1968; Clark et al. 1982). The early inflammatory cells release additional signals that can attract and activate fibroblasts, macrophages, and blood vessel endothelial cells. These cells infiltrate the wound site and form a specialized stroma called granulation tissue, which facilitates reepithelialization, helps contract the wound, and is later remodeled to form the scar.

Although many different cell types are present at the wound site, and dozens of signaling molecules, receptors, matrix proteins, and proteases are known to be expressed during the healing process (Martin 1997; Singer and Clark 1999), their roles in the process have been difficult to establish. This difficulty is due to the cellular and molecular complexity of wound healing and the challenges in manipulating wound gene expression and function in vivo. Hence, models of gene function in wound healing derive primarily from results of gene expression studies at wound sites, application of exogenous gene products to wounds, and studies in simple cell culture models such as keratinocyte monolayers. Analyses of wound healing defects in mouse knockouts of candidate genes have also begun to provide insight into the genes' roles in the process (Werner et al. 1994; Romer et al. 1996). However, some of the genetic results challenge fundamental aspects of the prevailing models (Ashcroft et al. 1999; Drew et al. 2001; Martin et al. 2003).

The establishment of simpler, more tractable genetic systems to study wound healing could allow systematic genetic dissection of the process in vivo and complement studies in vertebrates and clinical settings. Over a half century ago, Wigglesworth demonstrated that the large hemipteran insect Rhodnius prolixus has a robust wound healing response (Wigglesworth 1937). He characterized the response by light microscopy and described the proliferation and spreading of epidermal cells and the accumulation of blood cells (hemocytes) at the wound site. Since this pioneering work, only a few follow-up studies have appeared (Lai-Fook 1966, 1968). There has been little work on other insects aside from a number of studies of wound healing during imaginal disc and leg regeneration (Reinhardt et al. 1977; Truby 1985; Bryant and Fraser 1988) and the recent discoveries that *Drosophila* embryos undergo a scarless wound healing process involving actin cable formation and filopodial extension (Kiehart et al. 2000; Wood et al. 2002) and that wounded adult cells activate the Jun N-terminal kinase (JNK) signaling pathway (Ramet et al. 2002; see below). Some attention has also focused on melanization, the formation of a heteropolymer of orthoquinones generated by phenoloxidase-catalyzed oxidation of mono- and diphenols (Wright 1987) that accompanies certain infections, tumors, and wound healing (De Gregorio et al. 2002; Ligoxygakis et al. 2002).

We set out to investigate wound healing in Drosophila melanogaster because of the powerful genetic and genomic approaches available in this organism. These approaches have elucidated the molecular pathways that control many developmental and physiological processes. For example, genetic studies revealed a prominent role for a JNK signaling pathway in *Drosophila* dorsal closure, a developmentally programmed spreading of the embryonic epidermis (Noselli and Agnes 1999). This process resembles epithelial spreading during vertebrate wound healing, and indeed this similarity and the expression patterns of JNK pathway transcription factors near wounds (Verrier et al. 1986; Martin and Nobes 1992) prompted two recent genetic studies of JNK pathway activity in adult wound healing (Ramet et al. 2002; Li et al. 2003).

In this paper, we describe the cellular events and genetic requirements of epidermal wound healing in *Drosophila* larvae. A simple puncture wound assay was developed, and we use it to show that a plug rapidly forms at the wound site and subsequently melanizes to form a scab. We describe how epidermal cells surrounding the plug orient toward it and fuse to form a syncytium, and how the cells spread along and through the plug to reestablish epithelial continuity. We then use JNK pathway reporters and genetic analysis to demonstrate the induction and function of the JNK pathway in the process, and we use mutants that block scab formation, and a scabless wounding procedure, to elucidate the function of the scab. The results demonstrate that the cellular responses of wound healing are under separate genetic control, and that they are coordinated by multiple signals emanating from the wound site, including a negative feedback signal between scab formation and the JNK pathway. This establishes a tractable genetic system to study postembryonic wound healing, and the cellular and molecular parallels with vertebrate wound healing suggest that some of the fundamental steps in the process are evolutionarily conserved.

## **Results**

### **A Larval Epidermal Wound Healing Assay**

**Figure 1:**
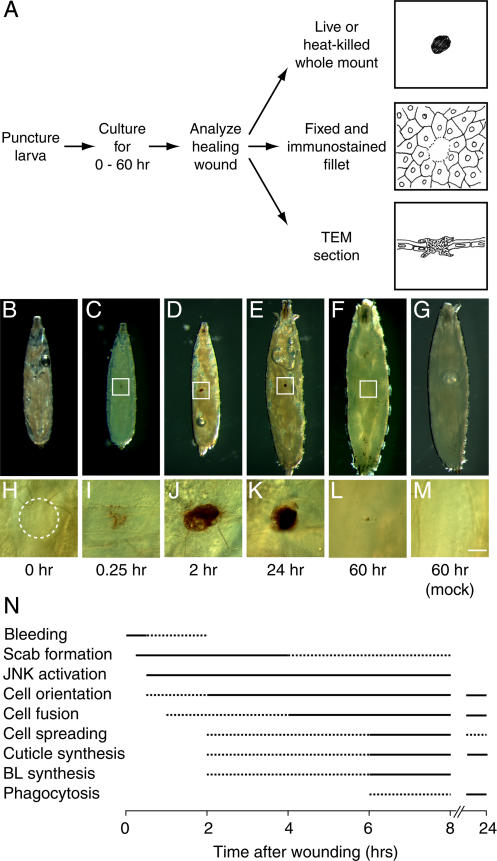
Scab Formation and Resolution during Puncture Wound Healing (A) Puncture wound assay. L3 larvae are punctured at the dorsal midline with a 100-μm diameter pin; they are then cultured and the healing wounds analyzed as shown. (B–E) Photomicrographs of heat-killed L3 larvae before wounding (B) and at the indicated times after wounding (C–E). Note larval growth during wound healing. Anterior is up. (F) L2 larva wounded as above and analyzed in L3, 60 h after wounding. Wounding in L2 allows visualization of late stages of wound healing without the complication of pupariation, which begins about 48 h after wounding in the standard L3 assay. (G) A mock-wounded L2 larva visualized 60 h after wounding. Note that it and the wounded larva (F) grew to a similar extent. (H–M) Close-up images of (B–G) showing unwounded cuticle (H and M) or wound sites (I–L, boxed regions in C–F) to show detail of scab. Micrographs are of living larvae taken shortly before the corresponding images of the whole heat-killed larvae above. Bar, 500 μm (for B–G), 50 μm (for H–M). (N) Timing of wound responses. Solid lines, time response was most often observed; dashed extensions to left, time response was occasionally observed; dashed extensions to right, time response was diminishing; BL, basal lamina.

**Figure 3:**
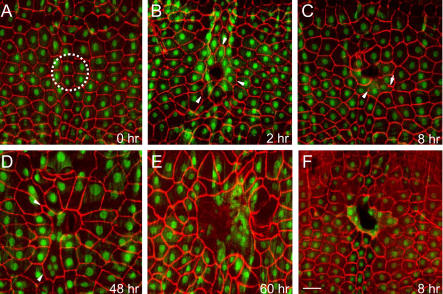
Epidermal Cell Orientation and Fusion around Puncture Wounds (A–E) *w; UAS-GFP.nls/+; A58-Gal4/+* larvae that express GFP (green) in epidermal cell nuclei were mock-wounded (A) or puncture wounded (B–E), cultured for the indicated time, filleted open, fixed, and immunostained for Fasciclin III (red) to label the basolateral surface of the cells. (A) Pre-wounding. Dashed circle, size of the 100-μm pin used for wounding. (B) 2 h postwounding. Some cells at the wound margin have elongated and oriented toward the wound (arrowheads). (C) 8 h postwounding. Cells at the wound margin have begun fusing to form a syncytium. Note the syncytium with four nuclei that contains a partially degraded, radially-oriented membrane domain (arrow) and scattered puncta of Fasciclin III staining in the cytoplasm (arrowhead) that may be membrane breakdown intermediates. (D) 48 h postwounding. The central syncytium contains ten or more nuclei, some of which are located in extensions (arrowheads) that may represent recent fusions of peripheral cells with the central syncytium. Other peripheral cells have oriented toward the syncytium but not fused with it. (E) 60 h postwounding. A large syncytium with more than 30 nuclei. (F) 8 h postwounding. Larva was treated as above but immunostained for Coracle (red), a septate junction component. The central syncytium contains nine nuclei. Bar, 50 μm.

A puncture wounding procedure was developed in which early third instar (L3) *Drosophila* larvae were lightly anesthetized and then stabbed with a 0.1-mm–diameter steel needle, about the size of six epidermal cells (Figure 1; see also Figure 3A). To ensure reproducibility, larvae were always stabbed at the dorsal midline halfway between the hair stripes of abdominal segments A3 or A4. Wounding did not cause a developmental arrest, because the wounded larvae continued to grow and pupariated 48 h after wounding, similar to mock-wounded controls (Figure 1B–1G), and 90% or more of the wounded larvae survived the procedure (see below). We then analyzed the major morphological, cellular, and molecular events of healing (Figure 1N) by visualizing wounds at different stages of healing in live and heat-killed whole-mount larvae, in histochemically or immunostained larval fillets, and in sections through wounds that we examined by transmission electron microscopy (TEM) (schematized in Figure 1A).

### **Bleeding and Scab Formation at the Wound Site**

**Figure 2:**
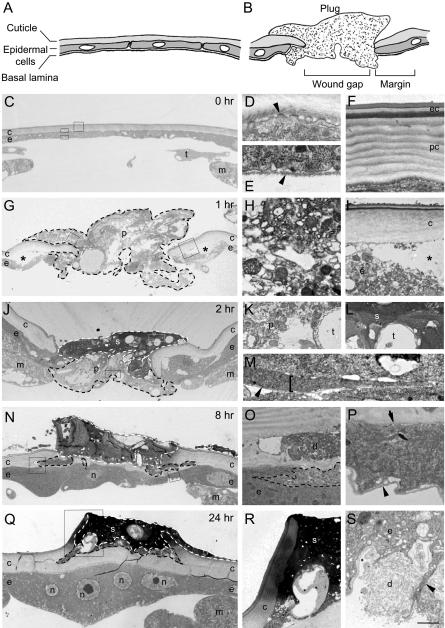
Ultrastructural Analysis of Puncture Wound Healing (A) Schematic of unwounded epidermis showing the cell monolayer, its apical cuticle lining, and basal lamina. White ovals indicate nuclei. (B) Schematic of recently wounded epidermis showing a plug of cell debris in the wound gap. Cells and ruptured cuticle at the wound margin are shown. (C–S) TEM sections of unwounded (C–F) and wounded (G–S) larvae at the times indicated after wounding. Transverse sections through each wound site are shown (C, G, J, N, and Q) along with close-ups of the boxed regions at right. c, cuticle; d, debris; e, epidermis; ec, epicuticle; m, muscle; n, epidermal nucleus; p, plug; pc, procuticle; s, scab; t, trachea (C) Pre-wounding. The epidermis and cuticle are intact. (D) Apical surface of epidermal cell showing villi (arrowhead) that secrete cuticle. (E) Basal surface. Arrowhead, basal lamina. (F) Epidermal cuticle. The epicuticle (top three layers) overlies the striated procuticle layer. (G) 1 h postwounding. The epidermis and cuticle are discontinuous but the gap is filled with a plug (outlined by dashed line) of cell debris. The epidermis has partially separated from the cuticle beyond the wound margin (asterisks). (H) The plug contains highly vesiculated cell debris. (I) The epidermis separating (asterisk) from overlying cuticle appears vesiculated and is presumably necrotic. (J) 2 h postwounding. The outer portion of the plug has melanized to form an electron-dense scab (outlined by white dashed line). The epidermis and cuticle are still discontinuous. (K) Debris, including a necrotic trachea, in the plug. The plug is not bounded by a membrane or basal lamina. (L) Portion of scab showing melanized debris and trachea. (M) Close-up of a lamellipodium (bracket) extending into a plug at the outer edge of another 2-h wound. Note basal lamina (arrowhead) along the lamellipodium. (N) 8 h postwounding. The epidermis has migrated across the gap to reestablish continuity, and has secreted new cuticle beneath the scab. (O) A region of epidermal cell cytoplasm near wound plug debris contains vesiculated material (outlined by dashed line) that is probably phagocytosed debris. (P) The newly established epidermis under the wound has a continuous basal lamina (arrowhead) and apical villi (arrow) secreting cuticle. (Q) 24 h postwounding. The new cuticle underlying the scab is thicker and the scab is more electron dense. Four nuclei in close apposition are in a syncytium because there are no membranes separating them. (R) Portion of scab and old cuticle. Note that cuticle in contact with the scab is melanized. (S) Cytoplasmic extension (arrowhead) engulfing debris at the basal surface of the epidermis of another 24-h wound. Bar, 10 μm (C, G, J, N, and Q), 0.33 μm (D and E), 0.83 μm (F), 1 μm (H, M, and P), 2 μm (I and O), 1.67 μm (K and L), 4 μm (R), 1.25 μm (S).

Unwounded larvae have a semitransparent white cuticular surface with rare or no blemishes (see Figure 1B, 1G, 1H, and 1M). Beneath the cuticle is the epidermis (Figure 2), an epithelial monolayer that secretes the cuticle at its apical (external) surface and is lined by a basal lamina along its basal surface (Figure 2A, 2C–2F). Immediately after puncture wounding, a variable amount of blood (hemolymph) escapes from the wound site (data not shown). Within 10–15 min, the wound site begins to darken (see Figure 1C and 1I) and a plug forms in the gap (Figure 2B and 2G). The plug is composed of debris, presumably the remnants of necrotic cells damaged by wounding that are disorganized and highly vesiculated and not bound by a cell membrane or basal lamina (Figure 2H and 2K). The plug may also contain blood coagulation products (see Discussion).

Over the next 24 h, the outer part of the plug is converted into a scab. This part of the plug becomes electron dense (Figure 2J and 2L) as the scab enlarges and darkens (see Figure 1D, 1E, 1J, and 1K), presumably due to a melanization reaction. Melanization affects all of the external structures at the wound site including the debris, the edges of the damaged cuticle (Figure 2N, 2Q, and 2R), and even entrapped tissues such as tracheae (Figure 2L).

By 2 or 3 d after wounding, debris is cleared, the scab resolves, and the exterior of the animal resumes a nearly normal appearance (see Figure 1F and 1L). Epidermal cells that grow back across the wound gap (see below) appear to participate in debris clearance, because they extend processes that engulf the debris (Figure 2S) and contain within their cytoplasm vesiculated material resembling debris (Figure 2O). Other components of the plug and scab may be degraded extracellularly or passively shed from the wound site.

### **Epidermal Cells Orient toward the Wound and Fuse to Form a Syncytium**

The response of epidermal cells to wounding was examined in transgenic larvae in which epidermal cell nuclei were labeled with green fluorescent protein (GFP) and cell membranes were immunostained for the basolateral membrane marker Fasciclin III or the septate junction protein Coracle (Figure 3). Epidermal cells at the wound site underwent two dramatic morphological changes in the several hours following wounding. First, beginning about a half hour after wounding, cells at the wound margin began to elongate and orient toward the wound, often tapering toward the wound site (Figure 3B). Second, these cells fused with each other to form a syncytium. Normally, epidermal cells are mononuclear (Figure 3A). However, as early as 1 h after wounding, the radially oriented plasma membrane domains (parallel to the long cell axis) began to break down as the circumferential domains joined, creating multinucleate cells around the wound. This can be seen in the Fasciclin III and Coracle stains, which showed incomplete (Figure 3C and 3) or absent (Figure 3C–3E) radial domains of plasma membrane staining; the loss of these membrane domains was sometimes accompanied by scattered puncta of staining in the cytoplasm, which may be membrane breakdown intermediates (see Figure 3C). TEM analysis confirmed the absence of plasma membrane between epidermal nuclei beneath and adjacent to the wound site (see Figure 2Q). Syncytia were nearly always present by 4 h after wounding.

As healing progressed, the polarization and fusion of epidermal cells spread outward from the wound. As cells bordering the wound fused, the more-peripheral cells just beyond the syncytium began to polarize toward the wound (see Figure 3D). Some of these cells apparently proceed to fuse with the central syncytium, because the average number of nuclei per syncytium increased over the 2 d following wounding, creating a large syncytium with as many as 30 nuclei surrounding the wound (see Figure 3E). Epidermal cell or nuclear division do not contribute to growth of the syncytium, because neither was detected around the wound site or elsewhere in the epidermis by immunostaining for phosphorylated histone H3, a marker of condensed mitotic chromosomes, 4–24 h after wounding (data not shown). Highly asymmetrical syncytia like the one shown in Figure 3D probably represent cases in which a subset of polarized peripheral cells had fused with the central syncytium. Peripheral cells may fuse to each other before fusing to the central syncytium, because satellite syncytia separate from the central syncytium were occasionally observed.

### **Epidermal Cells Spread along and through the Plug to Reestablish Epithelial Integrity**

A key step in wound healing is the closure of the epidermal gap and reestablishment of epithelial integrity. By 2 h after wounding, the epidermis was still discontinuous but the breach was filled by the plug and developing scab (see Figure 2J). Ultrastructural studies showed that during the next 6 h, as the epidermal cells oriented toward the puncture site and fused to form a syncytium, they also spread along and through the plug, led by lamellipodial extensions (see Figure 2M), until epithelial continuity was reestablished (see Figure 2N). No multicellular actin cable indicative of the “purse string” closure mechanism of embryonic wound healing (Wood et al. 2002) was observed in the spreading cells (see Materials and Methods). A thin basal lamina was present along the length of the lamellipodia (see Figure 2M), suggesting that basal lamina is synthesized by the cells before or during their migration. Following reepithelialization, new cuticle was secreted (see Figure 2N–2P). By 24 h after wounding, a thick new cuticle layer was present that was continuous with the old cuticle at the wound margin (see Figure 2Q). Most of the wound plug debris ended up outside the new cuticle layer and eventually melanized to form scab (see Figure 2Q and 2R), although occasionally some was left beneath the epidermis and was later phagocytosed or degraded (see Figure 2S).

### **The JNK Pathway Is Activated in a Gradient and Promotes Reepithelialization**

**Figure 4:**
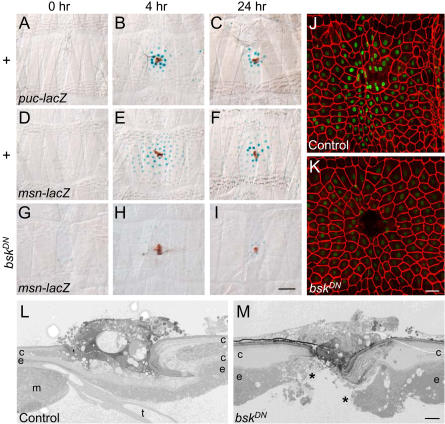
Induction and Function of the JNK Pathway around Puncture Wounds (A–C) Larvae carrying the JNK pathway reporter *puc-lacZ,* which expresses a nuclear β-galactosidase, were mock-wounded (A) or puncture wounded (B and C), and then cultured for the indicated times before staining with X-gal to visualize reporter activity (blue). There is little reporter activity in unwounded epidermis (A), but 4 h after wounding the reporter is expressed in a gradient emanating from the wound, with highest expression in the row of epidermal nuclei at the wound margin and decreasing levels in surrounding nuclei out to five cell diameters away. At 24 h (C), reporter expression has declined. (D–F) Larvae carrying the JNK pathway reporter *msn-lacZ,* treated as above. Wounding-induced reporter expression is seen out to seven cell diameters. (G–I) Larvae carrying *msn-lacZ* and *A58-Gal4* and *UAS-bsk^DN^* transgenes (to inactivate the JNK pathway in larval epidermis), treated as above. Reporter induction is inhibited, but the scab forms normally. (J and K) Larvae carrying *msn-lacZ* and either *UAS-bsk^DN^* alone as control (J) or *A58-Gal4* and *UAS-bsk^DN^* transgenes (K), wounded as above and analyzed 24 h later by immunostaining for Fasciclin III and β-galactosidase. Reporter induction is inhibited in (K), but epidermal cells have oriented toward the wound, and although nuclear β-galactosidase staining is faint, careful inspection shows that the cells closest to the wound have fused to form a syncytium. Syncytium formation was confirmed using the *A58-Gal4>UAS-GFP.nls* marker. (L and M) Larvae carrying *msn-lacZ* and either *UAS-bsk^DN^* alone as control (L) or *A58-Gal4* and *UAS-bsk^DN^* transgenes (M), wounded and analyzed 24 h later by TEM. Note that the epidermis in M has failed to spread across the wound gap and is still discontinuous (asterisks). No cuticle has been synthesized in the wound gap, but the cuticle flanking the wound appears thickened. Bar in (I), 100 μm (for [A–I]). Bar in (K), 50 μm (for [J and K]). Bar in (M), 5 μm (for [L and M]).

To elucidate the genetic control and interdependence of the cellular events of wound healing, we investigated the activity and function of the JNK signaling pathway in the process (Figure 4). The epidermal spreading in some ways resembles the epidermal spreading of dorsal closure, which depends on the JNK pathway. During dorsal closure, the mitogen-activated protein kinase kinase kinase kinase Misshapen (Su et al. 1998) is activated, triggering a phosphorylation cascade that ultimately activates the JNK Basket (Riesgo-Escovar et al. 1996; Sluss et al. 1996)*.* Basket phosphorylates the *Drosophila* Jun and *Drosophila* Fos transcription factors (Riesgo-Escovar and Hafen 1997), thus inducing expression of *puckered (puc),* which encodes a phosphatase that negatively regulates Basket, and other targets (Martin-Blanco et al. 1998). To test for JNK pathway activation in the larval puncture wound assay, we assayed expression of *lacZ* transcriptional reporters of *puc* and *misshapen (msn),* two genes induced by JNK pathway activation in other contexts (Martin-Blanco et al. 1998; Ramet et al. 2002).

In unwounded larval epidermis, there was little or no detectable expression of either the *msn* or the *puc* reporter (Figure 4A and 4D). However, within 1 h after wounding, expression of both reporters was readily detected in epidermal cells surrounding the wound, and by 4 h both exhibited robust expression (Figure 4B and 4E; unpublished data). The *msn* and *puc* reporters were induced in large, roughly symmetrical zones extending three to seven cell diameters out from the puncture site. Within each zone, the reporters were expressed in a gradient, with cells closest to the puncture site exhibiting the highest level of expression, suggesting that the reporters are induced by a signal emanating from the wound site. The zone of expression of the *msn* reporter was typically broader than that of *puc,* perhaps because it is more sensitive to the inducing signal. Expression of both reporters peaked between 4 and 8 h after wounding and declined thereafter, with expression restricting to cells closest to the wound (Figure 4C and 4F).

To determine the function of JNK pathway induction, we analyzed wound healing in larvae in which the JNK pathway was inactivated. Because null mutations in JNK pathway genes block dorsal closure and are embryonic lethal, we selectively inhibited the pathway in larval epidermis by expressing a dominant-negative form of Basket JNK *(upstream activation sequence-basket^dominant negative^ [UAS-bsk^DN^])* under the control of the *A58-Gal4* driver, an epidermal-specific driver that turns on early in larval development. *UAS-bsk^DN^* was used because it is the most potent JNK pathway inhibitor available (see Materials and Methods): it gave a severe dorsal closure phenotype and lethality when expressed in the embryonic epidermis using *e22c-Gal4* or*69B-Gal4* drivers. By contrast, larvae expressing *UAS-bsk^DN^* under control of the *A58-Gal4* driver were viable and active and did not display any morphological abnormalities, suggesting that the JNK pathway does not play a critical role in the larval epidermis under normal environmental conditions. However, following wounding, induction of the *msn* reporter was almost completely abolished (Figure 4G–4I), and the wound healing process was dramatically affected.

We analyzed the effect of JNK pathway inhibition on wound healing using the assays used for wild-type larvae. There were no detectable defects in the early steps in wound healing, including scab formation, epidermal cell orientation toward the wound, and epidermal cell fusion to form a syncytium (see Figure 4G–4K). However, ultrastructural analysis showed that reepithelialization was blocked or defective, with no cytoplasmic processes or only extremely fine or distorted processes and no new cuticle synthesis beneath the scab 16 h after wounding (Figure 4L and 4M; data not shown).

**Figure 5:**
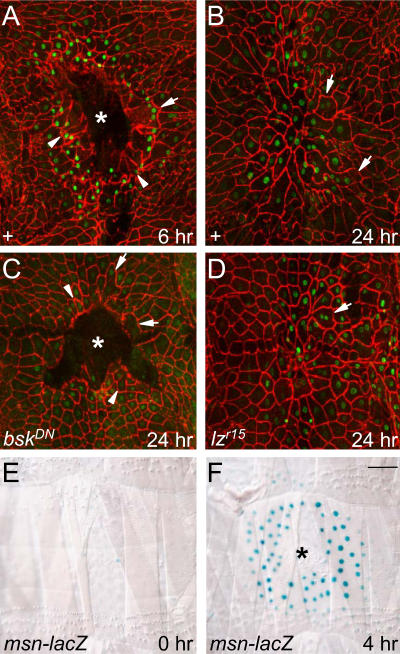
Cellular Responses and Genetic Requirements of Pinch Wound Healing (A–D) Larvae carrying the *msn-lacZ* reporter and the indicated transgenes or mutations were pinched with a forceps to abrade a region of dorsal epidermis but leave the overlying cuticle intact. Wounded larvae were cultured for the indicated times and immunostained for Fasciclin III (red) and β-galactosidase (green). (A) 6 h after pinch wounding. Note the large epidermal gap (asterisk) at the wound site. Some cells at the wound margin have elongated and oriented toward the wound (arrowheads). Others have fused to form syncytia (arrow). (B) 24 h after pinch wounding. The epidermis has spread to close the gap. Note disorganization of epidermis and syncytia (arrows) at site of healed wound. (C) An *A58-Gal4* and *UAS-bsk^DN^* larva 24 h after pinch wounding. Epidermal spreading is inhibited and a large wound gap remains (asterisk). However, cells at the wound margin still orient toward the wound (arrowheads) and fuse to form syncytia (arrows). (D) A hemizygous *lz^r15^* mutant larva 24 h after pinch wounding. *lz^r15^* blocks crystal cell development and scab formation at puncture wounds (Figure 6), but no defects are observed in pinch wound healing. (E and F) Larvae carrying *msn-lacZ* reporter were mock-wounded (E) or pinch wounded (F), cultured for 4 h, and stained with X-gal (blue). Wounding induces reporter expression in a gradient extending out four cell diameters. The gap (asterisk) lacks a scab. Bar, 100 μM.

To further test the requirement of the JNK pathway in reepithelialization, we analyzed larvae in which a portion of the epidermis was abraded by a nonpenetrating pinch wounding procedure (described further below) that leaves a much larger gap in the epidermis than does a fine puncture wound and hence provides a more rigorous test of wound reepithelialization (Figure 5). In control larvae in which the JNK pathway was not inhibited, the epidermis spread to close the gap, and full reepithelialization was evident within 24 h after wounding (Figure 5A and 5B). By contrast, in larvae in which the JNK pathway was inhibited, the epidermis did not spread, and a large gap remained (Figure 5C). We conclude that induction of the JNK pathway promotes spreading and reepithelialization of the larval epidermis but appears to be dispensable for other steps in wound healing, including scab formation, cell orientation, and cell fusion.

### **Crystal Cells Promote Scab Formation**

**Figure 6:**
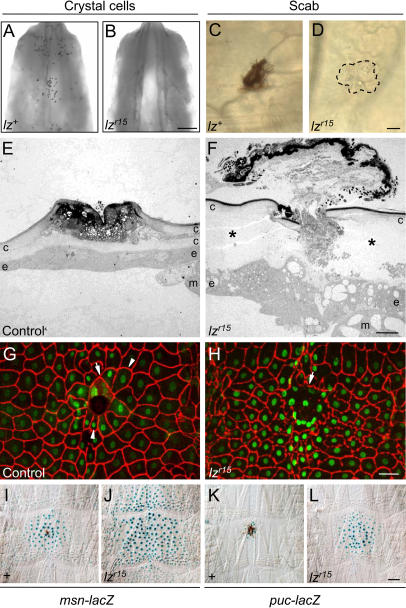
Effect of *lz* on Scab Formation and the Other Events of Puncture Wound Healing (A and B) Posterior of *lz^+^* (*w^1118^*) (A) and *lz^r15^* mutant (B) L3 larvae. Larvae were heated so crystal cells appear as tiny black dots. No crystal cells are apparent in the *lz^r15^* mutant. Bar, 200 μm. (C and D) Micrographs of control *lz^+^* (*w^1118^*) (C) and *lz^r15^* mutant (D) L3 larvae 4 h after puncture wounding. No scab is seen at the *lz^r15^* wound site (encircled). Bar, 50 μm. (E and F) TEM sections through 24-h–old puncture wounds of a control *lz^r15^*/+ heterozygote (E) and a hemizygous *lz^r15^* mutant larva (F), both carrying the *msn-lacZ* transgene. A consolidated, electron-dense scab has formed in the control larva (E), but only a diffuse plug with peripheral electron density is present at the *lz^r15^* hemizygous wound (F). The electron density of the *lz^r15^* plug might be due to residual melanization activity in the *lz^r15^* mutant. Although reepithelialization is complete in the *lz^r15^* mutant wound, the epidermis contains large vacuoles and abundant apical processes, and it is separated by a gap (asterisks) from the old cuticle and has not secreted new cuticle. Other 24-h *lz^r15^* mutant wounds analyzed had necrotic or discontinuous epidermis at the wound site (not shown). Bar, 10 μm. (G and H) Fluorescence micrographs of 20-h puncture wounds in control (G) and *lz^r15^* mutant (H) larvae carrying the *msn-lacZ* reporter that were treated as above and immunostained for Fasciclin III (red) and β-galactosidase (green). Epidermal cells at both control and *lz^r15^* mutant wounds have fused to form syncytia (arrows), and cells in the control are oriented toward the wound site (arrowheads). The orientation response of epidermal cells in the *lz^r15^* mutant is difficult to assess because cell borders out to six cell diameters away from the wound appear slack and wavy. Bar, 50 μm. (I–L) X-gal stains of 6-h–old puncture wounds of control *lz^+^* (I and K) or *lz^r15^* hemizygous mutant larvae (J and L) carrying either *msn-lacZ* (I and J) or *puc-lacZ* (K and L). Note the absence of scabs and the increase in reporter activity (blue) in *lz^r15^*. The basal level of reporter expression in unwounded epidermis was not increased in *lz^r15^* (not shown). Bar, 50 μm.

To investigate the role of the scab in puncture wound healing, we sought ways to block scab formation genetically (Figure 6). Crystal cells are a special type of blood cell that contain distinctive, crystal-like intracellular inclusions and have long been hypothesized to play a role in melanization responses such as those in scab formation (Rizki and Rizki 1959, 1984). The gene *lozenge (lz)* encodes a transcription factor required for development of the crystal cell lineage (Lebestky et al. 2000), and crystal cells are severely reduced or absent in *lz^r15^* homozygous or hemizygous larvae (Figure 6A and 6B). The *lz^r15^* mutant larvae failed to form a scab detectable by light microscopy (Figure 6C and 6D), and TEM analysis showed a diffuse plug at the wound site instead of the consolidated, electron-dense plug and scab that are normally present 24 h after wounding (Figure 6E and 6F). This defect in scab formation is likely due to the effect of *lz^r15^* on crystal cells, and not some other effect of the mutation, because scab formation was also inhibited in larvae homozygous for *Black cells (Bc)* (data not shown), a mutation that alters crystal cell morphology and eliminates serum phenoloxidase activity (Rizki et al. 1980). We conclude that crystal cells are required to consolidate and melanize the plug to form a scab during wound healing.

**Figure 7:**
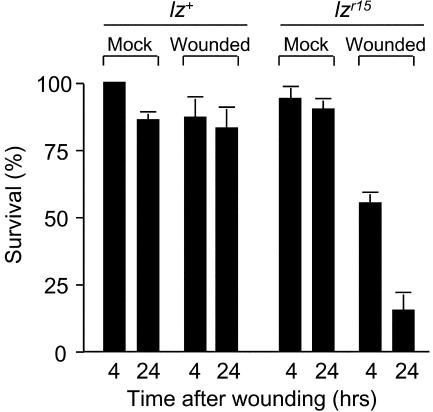
Effect of *lz* on Survival Following Puncture Wounding Control *lz^+^* (*w^1118^*) and *lz^r15^* mutant larvae were puncture wounded or mock-wounded and cultured for 4 or 24 h. The percentage of treated larvae alive and motile at each time is shown. Values are the average (± standard error of the mean) of three to six independent experiments with ten or more treated larvae per time point.

Untreated *lz^r15^* larvae were viable and active, but few survived the normal puncture wound procedure (Figure 7). By 4 h after wounding, only 55% of *lz^r15^* larvae were alive, and by 24 h only 15% survived, most of which were sluggish and flaccid. By contrast, 85% or more of the *lz^+^* control larvae survived the wounding procedure. Thus, scab formation is critical for healing puncture wounds.

### **The Scab Stabilizes the Wound Site and Prevents Superinduction of the JNK Pathway**

We next investigated the cellular events of wound healing in *lz^r15^* larvae, using the methods described above for wild-type and JNK pathway mutants, except that sharper pins were used for wounding to increase survival and allow analysis of the later stages of wound healing. Most of the cellular responses to wounding appeared to initiate in *lz^r15^* mutants, although they did not progress normally. Epidermal cell fusion occurred, but the syncytium often occupied a greater area than in control larvae (see Figure 6G and 6H). The surrounding epidermal cells also appeared to organize around the wound, but their cell borders were slack and wavy, even several cell diameters out from the wound, making it difficult to assess whether they had oriented toward the wound (see Figure 6H). A similar though less severe “wavy border” phenotype was observed in *Bc* mutant larvae. TEM analysis revealed that the epidermal cells up to 200 μm or more beyond the wound margin separated from the overlying cuticle around the wound (see Figure 6F). However, the detached cells extended numerous fine cellular processes in an apparent attempt to close the wound. Sometimes the edges of the punctured epidermis met to restore epithelial integrity, but in most cases they did not (see Figure 6F; data not shown).

The *lz^r15^* mutation also caused superinduction of the JNK pathway reporters. Although the basal expression level of the *msn* and *puc* reporters in unwounded epidermis was unchanged, both were expressed at higher levels and in an expanded zone around the wound site at 3, 6, and 24 h after wounding (see Figure 6I–6L; data not shown). A similar effect was observed in *Bc* mutants. Thus, scab formation limits induction of the JNK pathway around puncture wounds.

To further investigate the role of the scab in wound healing, a scabless wound healing procedure was developed. The larval cuticle was gently pinched with dissecting forceps, leaving the cuticle intact but abrading a patch of epidermal cells from its inner surface (see Figure 5A). Although these pinch wounds did not bleed or form scabs, the epidermal cells at the wound site underwent many of the same responses seen at puncture wounds. Many cells at the wound margin oriented toward the wound, and some fused with neighboring cells to form syncytia (see Figure 5A and 5B). Also, the *msn* reporter was induced in a gradient in the cells surrounding the wound (see Figure 5A and 5F), and the cells spread to close the wound gap within 24 h (see Figure 5B). Thus, each of the major epidermal cell responses to wounding can occur normally in the absence of a scab, provided the cuticle remains intact. Indeed, the primary function of the scab may be to restore integrity to the cuticle and wound site, because *lz^r15^*mutant larvae did not display any defects in the healing of pinch wounds: epidermal cells around the wound polarized and fused like in *lz^+^* controls, the JNK pathway reporters were induced at their normal levels and in their normal domain around the wound site, and the epidermal cells spread across the wound and healed with normal kinetics (see Figure 5D). Thus, the critical function of the scab appears to be to provide stability to the damaged cuticle and wound site, and the defects observed in the epidermal cell responses following puncture wounding of *lz^r15^* mutants most likely arise secondarily to the persistent instability of the wound site.

## **Discussion**

We established an epidermal wound healing assay in *Drosophila* larvae and elucidated the cellular events and genetic requirements of the healing process. Following puncture wounding, the damaged epidermal cells and their neighbors execute a series of responses that limit blood loss and restore integrity to the epidermis and overlying cuticle (see Figure 1N). Shortly after wounding, a plug forms in the wound gap. Over the next several hours, the outer portion of the plug melanizes to form a scab, and epidermal cells at the wound margin begin to elongate and orient toward the wound. They then fuse with each other to form a syncytium surrounding the wound. Subsequently, more-peripheral cells orient toward and fuse with the central syncytium. No proliferation of epidermal cells or actin cable formation was detected at the wound site. Instead, the epidermal cells surrounding the wound migrate along or through the plug to restore continuity of the epithelium and its basal lamina and cuticle lining.

**Figure 8:**
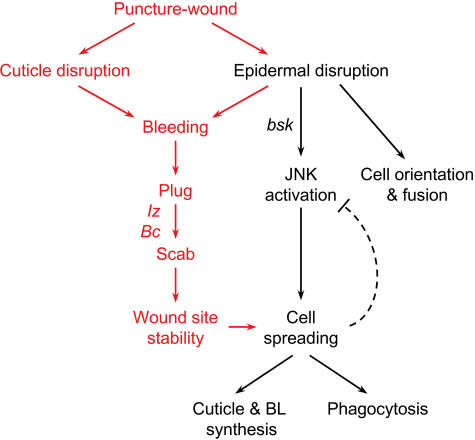
Model of the Cellular Events and Genetic Requirements of Larval Wound Healing Puncture wounding disrupts the epidermis and overlying cuticle and triggers the three parallel series of events shown, each with distinct genetic requirements. Plug and scab formation stabilize the wound site, which promotes epidermal cell spreading and suppresses JNK activation, perhaps by a negative feedback mechanism (dashed line). The *lz* and *Bc* genes promote scab formation, presumably by promoting crystal cell development and the production and secretion of serum melanization factors by these cells. The spreading epidermal cells synthesize cuticle and basal lamina, and they clear wound site debris by phagocytosis. Pinch wounding disrupts the epidermis but not the overlying cuticle and triggers only the events shown in black. However, cuticle and basal lamina synthesis and phagocytosis have not been examined in pinch wounds and are only inferred to occur from the puncture wound studies. Wounding may induce additional signals (not indicated) that attract blood cells (plasmatocytes) and tracheal branches.

Each of these responses—scab formation, epidermal cell orientation and fusion, and epidermal spreading and reepithelialization—occurs at characteristic times and positions during wound healing. However, our results suggest that these responses are under separate genetic control and are not contingently coupled (Figure 8). Scab formation is dependent on crystal cells and is inhibited by the *lz^r15^* and *Bc* mutations. Epidermal spreading and reepithelialization require *bsk* and JNK pathway activity, which is rapidly induced in epidermal cells surrounding the wound site. Epidermal cell orientation and fusion can proceed even in the absence of scab formation or JNK pathway activity. Although the different responses have distinct genetic requirements and can initiate independently of each other, we identified one important interaction between them. In *lz^r15^* and *Bc* mutants, reepithelialization initiated but was not always completed, and the JNK pathway was hyperinduced, implying that the scab normally facilitates reepithelialization and restrains JNK activation.

Below, we discuss the mechanisms and functions of each of these wound healing responses and the signals that trigger them, and suggest a mechanistic basis for the observed interaction between scab formation, reepithelialization, and JNK activation. We also compare wound healing in *Drosophila* with the related processes in mammals and speculate on their evolutionary relationship.

### **Formation and Function of the Scab**

The wound plug that forms shortly after puncture wounding contains cell debris, and it may also contain blood coagulation products like those identified in other arthropods (Nakamura et al. 1976; Barwig 1985; Geng and Dunn 1988) and recently in *Drosophila* (Scherfer et al. 2004). Over the next few hours the plug rapidly darkens and becomes electron dense, presumably the result of a melanization reaction. Although the nature and extent of melanin cross-linking to tissues has not been studied, it seems likely that the polymer links to wound plug components and cuticle to strengthen and stabilize the wound site.

Our results identify two important requirements for maturation of the plug and scab formation. One is crystal cells. The mutations *lz^r15^* and *Bc,* which block crystal cell development or function, inhibited scab formation at puncture wound sites. The effect was particularly striking in *lz^r15^* mutants: no scab was detected by light microscopy, and ultrastructural studies revealed only disorganized, amorphous debris where the scab normally forms. Because crystal cells are not commonly found at puncture wound sites (G. Fish, M. J. Galko, and M. A. Krasnow, unpublished data), these results support a model in which crystal cells promote scab formation by supplying serum factors such as prophenoloxidase that are necessary to form or consolidate the scab.

The other critical requirement for scab formation is a breach spanning both the epidermis and cuticle. In both puncture and pinch wounds, the epidermal layer is disrupted, but only puncture wounds formed scabs. The most obvious difference between the two types of wounds is that the cuticle layer remains intact after pinch wounding. This leads us to propose that scab formation is initiated by a signal generated or liberated by cuticle rupture, or by contact between serum and ruptured cuticle or air. One consequence of this would be local activation of prophenoloxidase by serine proteases that are present as inactive zymogens in insect cuticle (Ashida and Brey 1995; Jiang et al. 1998).

The scab appears to serve at least three functions in wound healing. One is to prevent exsanguination. *Drosophila* has an open circulatory system, so any rupture of both epidermis and cuticle will lead to blood loss. *lz^r15^* mutants did not form scabs and survived poorly after puncture wounding; the few surviving larvae appeared flaccid, suggesting continued blood loss from the wound. Although the wound plug likely provides a temporary stop to bleeding, scab formation appears necessary to form a stable hemostatic barrier. Second, the scab likely serves an immune function, which may also enhance survival upon puncture wounding. The orthoquinone precursors of melanin are cytotoxic to microorganisms (Nappi and Ottaviani 2000) and may guard against infection even before the scab matures to form a physical barrier to microbe entry.

The third function of the scab is to provide structural stability to the wound, which is critical for the next phase of wound healing, reepithelialization. This is inferred from the failure of reepithelialization following puncture wounding of *lz* mutants that are unable to form a normal scab. *lz* loss of function does not cause any intrinsic defect in reepithelialization, because reepithelialization of pinch wounds proceeded normally in the mutant. Also, the JNK pathway was activated in the wounded epidermis of *lz* mutant puncture wounds, and the cells at the wound margin appeared to initiate reepithelialization by extending processes into the wound gap. However, the epidermis did not always complete closure and seal the gap. These results suggest that when both epidermis and cuticle are disrupted, the scab is necessary to stabilize the wound gap to allow the epidermis to spread across and close it. In the absence of a scab, the JNK pathway is hyperinduced, epidermal cells at the wound margins separate from the overlying cuticle and extend abundant cytoplasmic processes, and a chronic wound ensues.

### **Epidermal Cell Orientation and Fusion**

Two intriguing cellular responses during wound healing are the orientation of epidermal cells toward the wound site and their subsequent fusion to form a syncytium. During orientation, epidermal cells at the wound margin lengthen along the axis radial to the puncture site and contract along the axis circumferential to it, with the part of the cell closest to the wound contracting most, imparting a characteristic taper. These cells then fuse by joining their circumferentially-oriented plasma membrane domains and eliminating their radially-oriented membrane domains that contact neighboring cells. This implies that epidermal cells are able to sense their location with respect to the wound and organize their cytoskeleton and plasma membrane domains with respect to it.

As wound healing proceeds, cell orientation and fusion typically spread to include more-peripheral cells, resulting in large syncytia with up to 30 nuclei at puncture wounds and smaller, scattered syncytia at pinch wounds. The occurrence of these responses in cells beyond the wound margin suggests that they are not a direct result of damage but rather are induced and oriented by a signal produced by wounding that can spread several cell diameters away from the wound.

The function of epidermal cell orientation and fusion may be to fit more cells around the wound and help seal off the wound site by eliminating intercellular spaces. This may be similar to the fusion of mammalian macrophages into multinucleate giant cells as they surround and engulf large foreign bodies (Chambers 1977). Indeed, like macrophages, the fusing epidermal cells appear to be phagocytically active, engulfing debris at the wound site. Although the close temporal and spatial relationship between epidermal cell orientation and fusion suggests that these responses are likely to be coupled, mutants that specifically block each process will be required to determine if they are contingently coupled or just coordinated by a common upstream signal.

### **Epidermal Spreading and Reepithelialization**

The most important cellular response for the long-term health of the animal is the restoration of epithelial integrity. However, spreading of the epithelium does not usually manifest until several hours after wounding. This allows time to induce the JNK pathway and activate the cell migration machinery in the epidermal cells, and to assemble a mature wound plug through or along which the cells move. Spreading appears to be an active process of epidermal cell migration, as no evidence of a purse-string closure mechanism or cell division was detected during spreading; instead, the earliest morphological manifestation of spreading was lamellipodial extensions, a hallmark of active cell migration, that course along and through the wound plug. Spreading likely requires a shift in the adhesion properties of epidermal cells from their normal tight association with the overlying cuticle to an affinity for the plug, and an ability to burrow through the plug.

Spreading also requires a signal at the wound site that induces the JNK pathway in surrounding cells and activates the cell migration machinery. This must be a local signal emanating from the wound site that can influence cells up to seven cell diameters away. The activating signal might guide the migrations across the wound gap, or the cells might spread randomly along the matrix until their movement is arrested by contact inhibition.

The main function of reepithelialization is to restore the normal barrier function of the epidermis. Indeed, the spreading epidermal cells ultimately secrete a thick layer of cuticle at their apical surface that displaces the scab, and they also supply the new basal lamina. The spreading cells also appear to play an important role in clearing wound site debris, as they were occasionally seen engulfing debris and often contained material resembling debris in phagosomes. Epidermal cells may share this scavenging role with plasmatocytes, circulating phagocytes recruited to wound sites after wounding (G. Fish, M. J. Galko, and M. A. Krasnow, unpublished data). Once reepithelialization is completed, debris is cleared, and the scab is sloughed or degraded, it is difficult to discern the old wound site by light microscopy. However, healing is not scar-free; the syncytium formed during healing persists and marks the wound site at least until metamorphosis begins. Occasionally, such syncytia are also seen in untreated larvae; these may be scars of naturally occurring wounds suffered earlier in larval life.

### **The Wound as a Signaling Center**

The results suggest that there are multiple signals induced by wounding that control and coordinate the different events of larval wound healing: a signal that initiates formation of the wound plug and scab, one that orients surrounding epidermal cells and induces them to fuse, one that activates the JNK pathway and epidermal cell migration, and one dependent on scab formation that suppresses the JNK pathway. There may also be signals that attract plasmatocytes to combat infection and tracheal branches to increase wound oxygenation (M. J. Galko, unpublished data).

These signals have distinct properties. One obvious difference is their range of activation around the wound. The signal that triggers plug and scab formation does so only at the epidermal and cuticular breach, whereas the JNK pathway activator influences cells up to seven cell diameters away. Some signals influence only the damaged cells and their neighbors, whereas others like the putative plasmatocyte and tracheal attractants must reach circulating cells and other tissues.

Some of the signals are likely to be diffusible molecules released by damaged cells. These could be intracellular components such as uric acid, histones, or heat shock proteins, all of which have been shown to be released by necrotic mammalian cells and are implicated as intercellular signals (Ohashi et al. 2000; Li et al. 2001; Scaffidi et al. 2002; Shi et al. 2003). They could also be more conventional signaling molecules like the fibroblast growth factors secreted upon vertebrate wounding (Werner et al. 1992).

Not all signals need be freely diffusible. Surface-bound signals could be sequentially propagated from one cell to the next, and some signals might be mechanical rather than chemical. Wounding appears to alter the tensile properties of the epidermis around the wound site (see Figure 6H), which could serve as a mechanical stimulus for some responses. This is an attractive idea for the control of JNK pathway activation, because changes in mechanical stress have been shown to activate JNK signaling in other cell types (Ingram et al. 2000; Kippenberger et al. 2000; Martineau and Gardiner 2001). Once reepithelialization is complete, tension could be restored, and signaling would diminish. Indeed, such a feedback circuit provides a plausible mechanistic basis for the inhibitory effect of scab formation on the JNK pathway (see Figure 8). In the absence of a scab, reepithelialization fails and tension is not restored, leaving the JNK pathway unconstrained.

It is not obvious how many distinct signals are generated by wounding, because individual signals might regulate multiple responses. A high priority now is to molecularly identify the signals and the mechanisms by which they control and coordinate the wound healing responses.

### **Comparison with Other Wound Healing Processes**

The healing of larval puncture wounds bears little resemblance to wound healing in the developing embryo, which occurs rapidly via actin cable assembly and filopodial extension by cells at the wound margin, and proceeds without scab formation (Kiehart et al. 2000; Wood et al. 2002). Despite the substantial structural differences between *Drosophila* and mammalian epidermis, embryonic wound healing appears similar to that in mammalian embryos, where it is also a rapid process involving actin cable formation but no apparent hemostatic or inflammatory response (Martin and Lewis 1992). Likewise, larval wound healing displays many similarities to postembryonic wound healing in mammals. Both processes commence with formation of a plug or clot that fills the wound gap. Both use the plug as a provisional substratum through which surrounding epidermal cells migrate. In both processes, the surrounding epidermal cells orient toward the wound site, become activated for migration, and spread through the plug in a similar manner—by extending lamellipodia and then their cell bodies into the plug until epidermal continuity is reestablished. The cells then redifferentiate to restore epidermal morphology. In addition, inflammatory cells are recruited to the wound in both processes, and the plug is remodeled to form a scab that is degraded or sloughed when repair and redifferentiation are complete.

Despite these general similarities, there are many specific differences between each parallel step in *Drosophila* and mammals. For example, the composition of the *Drosophila* plug and the mammalian clot probably differ, because clotting mechanisms in arthropods involve proteolytic cascades similar to those in mammals but different coagulogens (Nakamura et al. 1976; Barwig 1985; Geng and Dunn 1988; Scherfer et al. 2004). Also, *Drosophila* epidermal cells near the wound do not proliferate during reepithelialization as do their mammalian counterparts (Martin 1997). The cells surrounding a *Drosophila* wound fuse to form a syncytium, whereas mammalian epidermal cells remain distinct but dynamically rearrange their junctions with neighboring cells as they spread. Spreading *Drosophila* cells carry a basal lamina with them, whereas migrating mammalian epidermal cells detach from the basal lamina (Odland and Ross 1968; Clark et al. 1982). The most important difference may be the extent of cell recruitment to the wound site and subsequent remodeling of the plug, which are substantial in mammals but limited in *Drosophila*.

### **Evolution of the Wound Healing Response**

The similarities between *Drosophila* and mammalian wound healing responses prompt the question of whether these are homologous processes or the result of convergent evolution. Because there would likely have been strong selective pressure early in evolution for a wound healing response, we favor the idea that wound healing is an ancient process that evolved before the divergence of flies and mammals and subsequently diversified. Indeed, the parallels in the embryonic and postembryonic processes suggest that distinct embryonic and postembryonic wound healing mechanisms were already in place at the time of divergence.

If this evolutionary hypothesis is correct, then there should still be common molecular manifestations in *Drosophila* and mammals of the ancestral processes. Actin cable formation in embryonic wound healing may be one such manifestation, and the induction of JNK signaling pathways and their involvement in reepithelialization of postembryonic wounds may be another (Ramet et al. 2002; Li et al. 2003). Others may become apparent once the wound healing processes have been genetically dissected. The wound healing process described here, with its simple tissue architecture, streamlined response, and accessible genetics, provides a tractable system for identifying additional genes and fundamental mechanisms of wound healing.

## **Materials and Methods**

### 

#### **Fly strains and genetics**

The mutant *lz* ^r15^ is a molecular null allele (Daga et al. 1996). *Bc^1^* is a dominant mutation that was used in the homozygous condition (Rizki et al. 1980). The *msn-lacZ* allele was l(3)06946 (Spradling et al. 1999) and the *puc-lacZ* allele was l(3)A251.1 (Martin-Blanco et al. 1998); both are P[*lacZ, rosy^+^*] enhancer trap insertions in the respective loci that express a nuclear β-galactosidase; heterozygotes were used to monitor reporter activity. For analysis of *msn-lacZ* reporter activity in the *lz^r15^* mutant background, *lz^r15^, FRT18E/Y; msn-lacZ/+* hemizygous male larvae were compared to *lz^r15^, FRT18E/white [w]^1118^; msn-lacZ/+* heterozygous female siblings; similar comparisons were made for the *puc-lacZ* reporter. *w* ^1118^ was used as a control strain because most of the other strains employed carried a background *w^–^* mutation.

The Gal4/UAS system (Brand and Perrimon 1993) was used for protein misexpression. The *A58-Gal4* driver expresses the yeast Gal4 transcription factor throughout the larval epidermis beginning in L1 (A. Ghabrial, M. J. Galko, and M. A. Krasnow, unpublished data); *e22c-Gal4* (Lawrence et al. 1995) and *69B-Gal4* (Brand and Perrimon 1993) express Gal4 throughout the embryonic epidermis. *UAS-GFP-actin* (Verkhusha et al. 1999) was driven by A58-Gal4 to visualize actin dynamics within the larval epidermis*. UAS-GFP.nls* (Shiga et al. 1996) expresses a nuclear-localized GFP. *UAS-bsk^DN^* (Adachi-Yamada et al. 1999), *UAS-puc* (Martin-Blanco 1998), *UAS-Jra.bZip* (Kockel et al. 1997), and *UAS-kayak.bZip* (Zeitlinger et al. 1997) express different JNK pathway inhibitors. When crossed to the *e22c-Gal4* or *69B-Gal4* drivers, only *UAS-bsk^DN^* and *UAS-puc* gave a strong dorsal closure defect like JNK pathway mutants. To express Basket^DN^ in larval epidermis, female *w^1118^, UAS-bsk^DN^, UAS-bsk^DN^/w^1118^; msn-lacZ, A58-Gal4 /+* larvae (and sibling males lacking the *w^1118^* chromosome) were used. To express Puckered, *w^1118^; UAS-puc/ msn-lacZ, A58-Gal4* larvae were used. Larvae of the same genotypes but lacking *A58-Gal4* served as controls.

#### **Wounding assays**

Animals were reared on standard cornmeal-dextrose fly media at 25 °C. L3 larvae were rinsed with water, lightly anesthetized with ether, and then visualized under a stereomicroscope and impaled with a 0.1-mm steel needle (Fine Science Tools, Foster City, California, United States) at the dorsal midline between the hair stripes of abdominal segment A3 or A4. Typically, the needle pierced through the larva but only the entry wound was analyzed. After wounding, larvae were rinsed and returned to fly media in 1-dram vials and cultured at 25 °C. For experiments depicted in Figure 7, care was taken to select both larvae and wounding pins of uniform size because larval survival following wounding is significantly influenced by these variables. For pinch wounds, L3 larvae prepared as above were pinched with #5 dissecting forceps (Fine Science Tools) at midbody on the dorsal side for approximately 10 s and then cultured as above. Mock-wounded control larvae were prepared and cultured as above, except that needle impalement and pinching were omitted. Incisional wounds were not analyzed because incision caused early L3 larvae to burst and die.

#### **TEM**

Larvae were dissected at 4 °C in EM fixative (3% glutaraldehyde, 2% paraformaldehyde, and 2.5% dimethylsulfoxide in 0.2 M sodium phosphate buffer [pH 7.2]) and pinned ventral side up on a Sylgard (Dow Corning, Midland, Michigan, United States) surface. A ventral incision along the length of the animal was made with dissecting scissors, and the four corners of the epidermis were stretched with forceps and pinned to the surface. Internal tissues were removed, and the epidermis was fixed an additional 15 min at room temperature and then trimmed to a flat piece of epidermis surrounding the wound. Tissue samples were incubated for 1 h at 4 °C in 1% osmium tetroxide, stained overnight at 4 °C in 0.5% uranyl acetate, dehydrated through a graded series of ethanol concentrations and propylene oxide, and embedded in EMbed 812 (Electron Microscope Sciences, Hatfield, Pennsylvania, United States) with N, N-dimethylbenzylamine, which was polymerized overnight at 55 °C. Transverse sections (75–90 nm) were cut through the wound site with a Leica Ultracut ultramicrotome (Leica, Wetzlar, Germany) and collected on formvar/carbon-coated 75 mesh copper grids and stained for 20 s in supersaturated uranyl acetate:acetone (1:1) followed by 0.2% lead citrate for 3–4 min. Specimens were observed with an 80-kV beam on a JEOL TEM-1230 microscope (JEOL, Peabody, Massachusetts, United States), and images were captured on a Gatan Multiscan 791 digital camera (Gatan, Pleasanton, California, United States).

For TEM analysis of *lz^r15^* mutants, there was no scab to mark the wound site, so *lz^r15^, FRT18E/Y; msn-lacZ/+* larvae were used and stained with X-gal (5-bromo-4-chloro-3-indolyl--D-galactopyranoside) (see below) to locate the wound. Wounded larvae were dissected in phosphate-buffered saline (PBS), fixed in 2% glutaraldehyde for 15 min at room temperature, and stained with X-gal as described below, except the staining solution lacked Triton X-100 and contained 20 mM K_4_[FeII(CN)_6_], 20 mM K_3_[FeIII(CN)_6_], 2 mM MgCl_2_, and 0.2% X-gal in PBS. The propylene oxide dehydration steps during TEM sample preparation were omitted to preserve the X-gal reaction product.

#### Histochemistry and immunohistochemistry

For β-galactosidase histochemistry, larvae carrying *lacZ* transgenes were dissected open in PBS, fixed for 15 min at room temperature with cold 2% glutaraldehyde, rinsed with PBS, and then stained at room temperature for 6 h *(puc-lacZ)* or 2 h *(msn-lacZ)* in 150 mM NaCl, 10 mM Na_2_HPO_4_, 3 mM K_4_[FeII(CN)_6_], 3 mM K_3_[FeIII(CN)_6_], 1 mM MgCl_2_, 0.1% Triton X-100, and 0.2% X-gal.

For immunostaining, primary antibodies were anti-Coracle monoclonal antibodies 9C and C61516B (Fehon et al. 1994) (1:500 dilution), anti-Fasciclin III monoclonal antibody 7G10 (Patel et al. 1987) (1:50 dilution), and rabbit anti-β-galactosidase serum (Roche, Basel, Switzerland) (1:150 dilution) preadsorbed against *Drosophila* embryos. Secondary antibodies (Jackson Immunoresearch, West Grove, Pennsylvania, United States) were goat anti-mouse IgG-Cy3 (1:1000 dilution) and goat anti-rabbit IgG-FITC (1:300 dilution). Samples were blocked in PHT buffer (Ca^++^/Mg^++^-free PBS containing 1% heat-inactivated normal goat serum and 0.3% Triton X-100) for 1 h or more and then incubated overnight at 4 °C with primary antibody diluted in PHT. Samples were washed with fresh PHT at least six times for 1 h at room temperature, incubated overnight at 4 °C with secondary antibody, and washed as before. Samples were mounted in 70% (v/v) glycerol or Vectashield (Vector Laboratories, Burlingame, California, United States) mounting medium and observed with a Bio-Rad confocal microscope (Bio-Rad, Hercules, California, United States). Cy3 and FITC channels were sequentially excited and captured for each specimen; a Z-series of optical sections was collected and merged to avoid loss of out-of-plane information due to tissue wrinkling.

## Supporting Information

### Accession numbers

The GenBank (http://www.ncbi.nlm.nih.gov/) accession numbers for the genes discussed in this paper are *bsk* (NM_164901), *Jra* (NM_165739), *kay* (NM_170427), *lz* (NM_078544), *msn* (NM_079940), and *puc* (NM_079549).

## Abbreviations

*Bc*: *Black cells*
*bsk*: *basket*
DN: dominant negative
GFP: green fluorescent protein
JNK: Jun N-terminal kinase
L[number]: larval instar [number]
*lz*: *lozenge*
*msn*: *misshapen*
PBS: phosphate-buffered saline
*puc*: *puckered*
TEM: transmission electron microscopy
UAS: upstream activation sequence
*w*: *white*
